# Stand Together by Staying Apart: Extreme Online Service-Learning during the Pandemic

**DOI:** 10.3390/ijerph19052749

**Published:** 2022-02-26

**Authors:** Christian Compare, Cinzia Albanesi

**Affiliations:** Department of Psychology “Renzo Canestrari”, University of Bologna, 40127 Bologna, Italy; cinzia.albanesi@unibo.it

**Keywords:** extreme online service-learning, mixed-method, longitudinal design, democratic competencies, sense of community responsibility, COVID-19 lockdown

## Abstract

Service-Learning (SL) is an experience that allows students to (a) participate in activities co-designed in partnership by universities and local organizations and (b) reflect on the service activity in such a way as to gain an enhanced sense of responsibility. These experiences represent significant ways to meet and experience real-world contexts for students. The COVID-19 pandemic required Higher Education Institutions to rethink and shift in-presence courses to online platforms. This transition included SL courses as well. This study aimed to explore the responsibility and democratic dimensions elicited by an extreme online Service-Learning (XE-SL) experience and the perceptions of engaging in exclusive online service activities with local communities during the COVID-19 Italian national quarantine. A qualitative driven mixed-method longitudinal approach was chosen to triangulate qualitative (reflexive journal) and quantitative (pre-post questionnaire) data from 20 university students. The findings shed a positive light on the capability of XE-SL to promote a sense of responsibility, civic engagement, and the acquirement of democratic and transferrable competencies, such as perspective-taking, adaptability, cultural background respect, global mindedness, teamwork, leadership, communication, creativity, and organizational competencies. Reflection, connection, and being agents of change for the community were perceived as the major assets of the XE-SL experience, while adapting face-to-face SL experiences to exclusively online activities evoked ambivalent feelings in students. The study suggests a rethinking of the design XE-SL and other forms of eSL with the inclusion of more structured interactive activities within community contexts to favor students’ sense of connection to the community organizations or NGOs.

## 1. Introduction

The COVID-19 pandemic caused abrupt changes to teaching and learning. In the immediate aftermath of the COVID-19 outbreak, the Italian Government required the suspension of in-presence learning activities at diverse levels of education. The transition from face-to-face to emergency remote teaching—ERT [1] was challenging for all levels of education as it was rapid and unexpected: the limited experiences of the application of digital technologies, as well as the habits, to heavily rely on traditional methods, exacerbated the negative impact of this forced transition in secondary schools [2]. At the higher-education level, the transition to ERT was also challenging. Although many universities were more acquainted with technological resources (i.e., digital platforms for synchronous and asynchronous interactions), faculty and instructors had to evolve their courses toward online teaching in record time. In different circumstances, the same online shift would have required years to take place [3]. The online shift during the pandemic also applied to Service-Learning courses.

### 1.1. Service-Learning

Service-Learning is an experiential approach aimed at developing personal, professional (subject-specific), and civic and democratic competencies of students. It integrates meaningful community service or engagement into the curriculum, offering students academic credit for the learning that derives from active participation within the community and work on a real-world problem. Based on developing a sense of community responsibility (SOC-R) [4], SL should support students’ capacity to act effectively and responsibly for the collective well-being. Reflection and experiential learning strategies underpin the learning process, and the service is linked to the academic discipline [5]. SL contributes to students’ “reality check” and competencies mobilization and development by offering the opportunity to articulate learning through reflections and engaging in real-world issues. Service-Learning benefits for students range from personal/transferable competencies (self-efficacy; adaptability) to democratic competencies (understanding of social issues) to disciplinary ones (academic performance) [6,7,8,9].

Nevertheless, defining transferable and democratic competencies is not an easy task. Transferable skills are also referred to as competence for life, key competence, and generic competence [10]. According to the Council of Europe [11], they identify knowledge, skills, and attitudes needed by all for personal fulfillment and development, employability, social inclusion, and active citizenship. The Council of Europe has also defined democratic competence within the Reference Framework of Competencies for Democratic Culture (RFCDC) [12]. They identify a set of attitudes, values, skills, and knowledge that need to be acquired by learners to participate effectively in a culture of democracy and live peacefully together with others in culturally diverse democratic societies and to feel a sense of belonging and make their positive contributions to the democratic societies in which they live. In line with the RFCDC, the OECD [13] (p. 4) promotes the acquiring of a global perspective in examining local, global, and intercultural issues, interacting successfully and respectfully with others while taking responsible action toward collective well-being. Building on the proposed global competence framework, the OECD stresses the relevance of competencies assessment.

### 1.2. Democratic Competencies

Democracy is a form of governance responsive to the views of the majority, and that requires citizens’ commitment to participate actively in the public good. The Council of Europe’s White Paper on Intercultural Dialogue [14] points out that democratic and intercultural competencies are not acquired automatically by citizens but, instead, need to be learned and practiced. Education is uniquely positioned to guide and support learners—as citizens—in this and empower them. Educational Institutions can help learners develop in the acquirement of the competencies they need to become active and autonomous participants in democracy, intercultural dialogue, and society, giving them the tools to craft their own scopes while respecting human rights, the dignity of others, and democratic processes [12].

According to Barrett [15] (p. 9), democratic competence can be defined as “the ability to select, activate, organize, and apply relevant psychological resources in order to respond appropriately and effectively to the demands, challenges, and opportunities that are presented by democratic situations”. This competence should be perceived in dynamic terms as competent individuals will mobilize and use psychological resources dynamically and fluidly, self-regulating their behavior and adjusting it according to situational circumstances.

Democratic competencies include skills, knowledge and understanding, and values and attitudes, regarded as essential for behaving appropriately and effectively in democratic and intercultural situations. Values and attitudes are considered psychological resources that can be activated, organized, and applied through behavior to respond appropriately and effectively in democratic and intercultural situations [12].

### 1.3. eSerivice-Learning

Even if the learning capacity of SL is embedded in hands-on, experiential learning in the community, online SL learning has been developed to deal with those situations in which face-to-face SL is not practicable. Waldner et al. [16] identified different ways of online SL. They included (a) hybrid forms where: the instructional part is online and the service part is offline, or vice versa (type I and type II, respectively); and the instructional part and service are partially online and partially offline (type III); and (b) an extreme online SL type (XE-SL; type IV), where both instructional and service parts take place online.

### 1.4. eSerivice-Learning—What Are the Benefits for Students?

Many scholars claim that the benefits of eSL and XE-SL for students are equivalent to offline “traditional” SL [17], while others identify additional benefits, including accessibility, cost reduction, and capacity to reach diverse communities [18].

Among benefits, eSL is reported to stimulate students’ teamwork competency [19], social justice and civic attitudes [20], learning engagement, gradual increment in emotional engagement during XE-SL experiences, starting from the initial uncertainty and mixed feelings to final positive emotions [21], and life satisfaction even during challenging situations such as the COVID-19 pandemic [22]. According to the literature, eSL can facilitate the application of textbook knowledge to solve actual problems while fostering more university-to-community relationships and extending the university’s reach with the geographic dispersion of the online student populations [23]. Moreover, removing geographical constraints is reported to facilitate (virtual) mobility, offering students the opportunity to develop global awareness of social issues [21,24].

However, with few exceptions, the literature on eSL benefits for students is anecdotal [16]. Faulconer [25] reviewed the papers published between 2010 and 2020 on type II and IV SL (where service takes place online) and found that few studies described how students were assessed on their eService-Learning activities. Only one reported using rubrics to evaluate transferable skills [10]. Faulconer [25] (p. 15) concluded that assessment was largely overlooked in the research of eService-Learning, possibly because it still represents a challenge in SL. She suggested using mixed-methods research to understand better how to evaluate and assess eService-Learning projects.

The problem of online teaching and learning assessment is not exclusively limited to eSL. In a systematic review of 262 studies conducted in 2020 during the “global online semester” to analyze students’ learning experiences during the pandemic, Bond et al. [26] highlighted how limited attention was devoted to learning outcomes assessment stressing the relevance of further exploration.

Outcomes-based assessment is imperative in programs such as Service-Learning, that build on university–community partnership. Learning outcomes, indeed, indicate to faculty, students, and the community the specific results expected from participation in the program [27] and shape the collaborative design of SL courses between faculty and community partners.

Moreover, given that the learning dimension of SL relies heavily on hands-on experience in the community, understanding how the online emergency shift affected the capacity of XE-SL to achieve its learning objectives (i.e., developing personal, transferable, and democratic competencies) is of utmost relevance.

Therefore, this paper aimed to contribute to the academic field by focusing on an XE-SL experience during the COVID-19 national quarantine in Italy. Intending to contribute to the academic discussion and better understand the specific aspects of exclusive online SL activities, we explored the responsibility and democratic dimensions elicited by the XE-SL experience and the perceptions of engaging in exclusive online service activities with local communities.

### 1.5. Study Purpose and Research Questions

The study aimed to explore the responsibility and democratic dimensions elicited by the XE-SL experience and the perceptions of engaging in exclusive online service activities with local communities. The research questions that guided the study are the following:

RQ1. What is the role of XE-SL in enhancing students’ sense of responsibility toward the community?

RQ2. How are the democratic competencies of students strengthened by the XE-SL experience?

RQ3. What are the limits and assets of XE-SL as perceived by students?

### 1.6. The Context

The introduction of SL at the University of Bologna started in 2015. In late 2016, a pilot experience was implemented, developing an SL lab for 30 Clinical Psychology master’s students. Since that time, SL has continued to grow [28], and since 2018, courses of SL have been included in the program catalog for the development of transferable competencies for students enrolled in any degree program of the university. To support the growth of SL experiences, the Department of Psychology established different partnerships with several local organizations in two different campus branches. The scope of the partnerships grew from one local partner and six SL projects (for one academic module) to 23 local partners and 24 SL projects (for three academic modules) in six years, involving approximately 80 students every year. At the beginning of 2020, 15 organizations located in two campus branches of the university were ready to host the students of the transferable competencies courses starting in late February. The service component of the courses was planned on-site, and the students were eager to go into the field. The COVID-19 outbreak required all courses to move online, including SL. Before the COVID-19 national quarantine, SL courses and modules were exclusively implemented and offered in face-to-face forms. Therefore, community partners were summoned to a meeting to inquire about the eventual availability and possibility of shifting their service online (rethinking also aims and activities). Only ten organizations were able to adapt their activities and host students online. Given its online format, some students decided to opt for more traditional courses. Twenty students started the SL courses by mid-March, with online service activities starting in mid-April.

## 2. Materials and Methods

### 2.1. Study Design

A mixed-method longitudinal approach was chosen for triangulation to assess the extent to which qualitative and quantitative findings corroborate each other [29]. The choice of this approach was also determined by the reported scarcity of mixed-method studies in SL research to stimulate additional insights and further understanding not gleaned from one approach alone [30].

**Figure 1:**
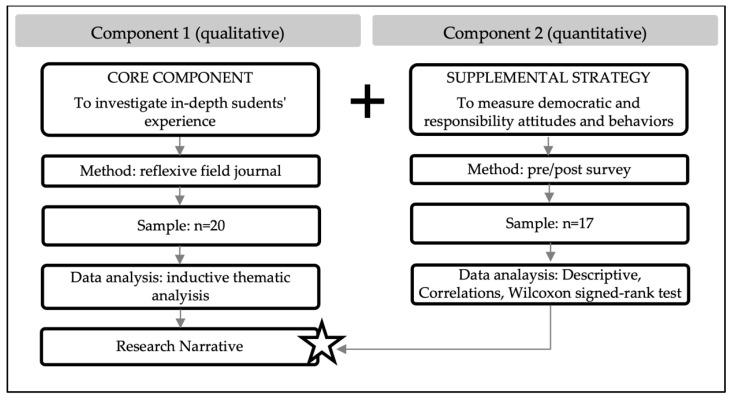
Simultaneous mixed-method design.

The study used a qualitative-driven, simultaneous mixed-method design, with one supplemental quantitative longitudinal component (Figure 1). Its theoretical drive was deductive [31]. Qualitative and quantitative data were collected concurrently, analyzed independently, then merged and integrated to create the research narrative, as outlined by Morse and Neihaus [32]. Qualitative data were used to investigate in-depth students’ experience, and quantitative data were used to expand the understanding of measurable democratic and responsible attitudes and behaviors and their trajectories in SL with pre- and post-data collections.

The qualitative component consisted of reflexive journals, and the quantitative component was a pre/post-experience survey of democratic competencies and the sense of community responsibility.

### 2.2. Participants

The participants were 20 university students enrolled in an XE-SL course for transferable competencies held online between March and June 2020. This was the only SL course offered at the University of Bologna during the COVID-19 national quarantine. Half of the participants (*n* = 10; 50%) were cis-gender women and the other half cis-gender men. The students’ academic fields of study were distributed as follows: 35% Engineering; 35% Behavioral and Social science (i.e., psychology, anthropology, and political science); 10% Business and administration; 10% Arts; and 10% Humanities (i.e., philosophy and literature). Most of them lived in the Northern regions of Italy (*n* = 16; 80%), 10% in the Central regions, and 10% in the Southern regions. Many participants (*n* = 12; 60%) declared to live in the area of the two campus branches in which the community organizations promoted their activities during the COVID-19 Italian lockdown. Students’ service activities within the community organizations were distributed as follows: 50% Social Services (e.g., elder people, blinded children); 25% Education (i.e., virtual school activities); 15% Organizational (e.g., web page make-over); 10% Healthcare (i.e., oncology awareness campaign).

### 2.3. Measures

Qualitative reflexive journals and quantitative pre-post course questionnaires were used to collect data.

The qualitative reflexive journal assigned to participants was composed of three sections and provided prompts to students. The sections were: (1) a general description of the SL project and the community organization; (2) accurate service activity report, reflections on the activities (e.g., positive or negative feelings, encountered people, the meaning of the service activity), and analysis of the effects of the experience on the learning dimension (e.g., acquired or deployed competencies, prejudice awareness); (3) final report with an analysis of the XE-SL experience, the relationship between the experience and the civic engagement dimension, and a final reflection on the awareness produced by the experience. Quantitative questionnaires included the following measures:

*Democratic Competencies*. To assess the democratic competencies, we used the attitudes and skills subscales of the Global Competence Questionnaire [13]: perspective-taking subscale, five items (α = 0.72; e.g., “I believe that there are two sides to every question and try to look at them both”); adaptability subscale, six items (α = 0.67; e.g., “I can deal with unusual situations”); respect for people from other cultural backgrounds subscale, five items (α = 0.74; e.g., “I give space to people from other cultures to express themselves”); global mindedness subscale, five items (α = 0.75; e.g., “I think of myself as a citizen of the world”). Answers were scored on a 5-point Likert scale, ranging from 1 (not at all) to 5 (completely).

*Personal Beliefs about Inequalities*. To measure respondents’ personal beliefs about inequalities, we adapted the Critical Consciousness Scale—Short form [33]. We included the Critical Reflection-Perceived Inequalities subscale in the survey, eight items (α = 0.96; e.g., "Poor people have fewer chances to get good jobs”). Answers were scored on a 5-point Likert scale, ranging from 1 (not at all) to 5 (completely).

*Sense of Community Responsibility*. To assess respondents’ sense of responsibility toward the community, we used the Sense of Community Responsibility Scale [34]. The scale consisted of six items (α = 0.66; e.g., “It is easy for me to put aside my own agenda in favor of the greater good of my community”). Answers were scored on a 5-point Likert scale, ranging from 1 (not at all) to 5 (completely).

### 2.4. Data Collection Procedures

Qualitative data were collected throughout the experience. Participants filled the reflexive journal during the experience. At the beginning of the course, students were asked to report after each service activity notes and reflections on the meaning of what they did, the competencies they deployed, and the learning dimension of the experience. Quantitative data were collected before and upon completion of the experience with a pre-post survey of close-ended items. The research was conducted following the European Code of Conduct for Research Integrity and the ethical guidelines of the Italian Association of Psychology. Students were informed of the purposes of the research and expressed written consent for their participation. The researchers clarified (together with the class instructor) that the pre-post survey was anonymous and that to preserve students’ anonymity, they had to insert a self-generated code to allow matching of the two questionnaires. Filling the questionnaire was part of the course requirement, but there were no mandatory questions in the survey, and students could skip all questions. Students were also informed that their perspectives were essential to evaluate course design, weaknesses, and strengths. Concerning reflexive journals, it was clarified with students, when presenting it as part of the course assignments, that they could have been shared with the research team in addition to the instructor, and in case they would be used for research purposes, they would be treated to preserve confidentiality and privacy. Students were aware that reflexive journals would be used to award the final grade for the course, as it was a tool intended to grasp students’ capacity to reflect on their experience critically. Given that there are many ethical concerns regarding the recruitment of university students as research subjects, including coercion and other forms of misconduct [35,36,37], the research team tried to be as transparent as possible and engaged in a reiterative process of confirming consent, particularly regarding the use of quotes of reflexive journals.

### 2.5. Data Analysis

Two datasets were produced (quantitative data and qualitative reflexive journals). Datasets were examined independently, then integrated through a narrative in a weaving approach following Fetters’ guidelines [38]. Qualitative analysis was performed with an inductive thematic analysis following the phases of thematic analysis as reported by Braun and Clarke [31]. The corpus of the reflexive journals was read and re-read to familiarize with the data, noting down initial ideas. Interesting features of the data were coded systematically with initial codes. Codes were then collated into potential themes. Subsequently, themes were reviewed, and coded extracts were reported in a spreadsheet. Lastly, a final analysis of selected extracts was performed. Four themes emerged from the thematic analysis: sense of responsibility, competence awareness, XE-SL assets and limits, XE specificity of the SL experience.

Quantitative analysis was performed using SPSS 26. Given the small sample, normality was checked for. The Shapiro–Wilk test of normality showed significant results; therefore, nonparametric tests were performed on the data. Descriptive statistics and correlations (Table 1) and the Wilcoxon signed-rank test (Table 2) compared scales scores between pre- and post-surveys.

## 3. Results

### 3.1. Sense of Responsibility and Competence Awareness: Qualitative Data

Data of the first and the second research questions were interrelated and are therefore presented together.

#### 3.1.1. Sense of Responsibility

Qualitative data on responsibility unfolded different perspectives on the inner meaning of being community-engaged. Participants reported how the experience helped them frame and reinforce their idea on community engagement, entirely focused on promoting change within the community and letting it grow. University as an institution scaffolded this civic learning by allowing students to join XE-SL.

“Community engagement means to devote our strength, our passion, our knowledge, and our time to others. Community engagement means considering others as an enrichment, helping people integrate and live better in society. Serving the community is a reciprocal act of change and societal growth. My university had a major role in starting this process for me. I wanted to be engaged with the community somehow for a long time. University helped me to get in touch with this perspective”. (EL_C).

Helping the community to grow and to contribute to positive change that can make people’s lives better was a relevant starting and ending point for students, i.e., a motivation and a final goal at the same time. Designing products, projects, and services for others was also perceived as a valuable lesson for students’ future as responsible practitioners.

“I’ve learned the relevance of realizing ideas and new products to make community life better. I think that I have consolidated my civic sense. I’m confident that it’ll be helpful in my working life. I want reciprocity and community engagement to be two essential principles in my future job”. (GA_B).

“I made something useful for someone, and I think this is one of the most gratifying things that you can receive when you serve the community”. (GR_C).

Participants reported how community organizations welcomed the deployment of their academic and personal knowledge, giving back new learning and the “precious gratitude” of community members.

“To serve the community means to offer my professional competencies, my knowledge, and my personal experiences to help a part of the community, that offers back a very powerful way to learn”. (VL_C).

“I put myself forward for helping the organization, especially with the elderly. I think that engagement means collaborating to create the common good, and that’s what I did. I made myself available to help elderly people with the use of technology, and I received back something precious: their gratitude”. (LD_B).

Community organizations’ capacity to host students positively and engagingly made participants feel valued.

“I didn’t expect to be so involved by the organization. Although we were just ‘rookies’ for the organization, we have been involved several times in decisions on issues to be addressed or activities to promote. I think it’s a great thing.”. (GA_B).

Sometimes, the experience poorly met the expectations of students. One participant shared that he encountered some difficulties during the XE-SL, disclosing how he transformed them into "fuel" to be more determined in achieving the project’s tasks and mission.

“I learned to transform moments of disorientation and disappointment in opportunities to be more motivated in working for this [XE-SL] project. This approach encouraged me to be even more determined”. (AB_C).

In another situation, by a reflective process over the meaning of the disappointment generated by the experience, one participant was able to share with the community organizations the critical aspects to ameliorate and rethink the project for future XE-SL students and the final users (i.e., elderlies).

“I cannot say that my contribution to society is inexistent or that my commitment is worth nothing. On the contrary, by experiencing the critical aspects of this project, I was able to share a reflection with the organization on what can be improved for improving the experience both for XE-SL students and users”. (MGS_B).

Strictly related to responsibility, the positive exchange and reciprocity seemed to be critical elements to raise and internalize a sense of responsibility for the community. The experience helped students to also reflect on their level of engagement and conclude that everyone can be an agent of change.

“I realized that I do too little for others, and I would like to do more. I understand now that everyone can help to make things better”. (CR_B).

Even if in its virtual form, human interaction helped students raise their sense of responsibility and reinforce a call for community actions. Moreover, the community dimension became a place to present themselves and their stories, a valuable repository of shared narrations.

“Receiving positive feedback from the children [target community] made me feel welcomed. During the meeting, I was able to make my modest contribution by sharing my story. This made me feel useful. Today my words are finally valuable for someone, which was important to me. I am fully aware that community starts from us as individuals; being updated on political, social, and economic issues, being active listeners of the needs that society brings, fighting for them, and promoting the recognition of fundamental rights and the empowerment of marginalized and underserved communities”. (AB_C).

The responsibility dimension also emerged concerning the pandemic situation. XE-SL represented an opportunity to start a reflection, considered a process to be still “rooted to the ground”, on solidarity and social responsibility in a broader sense.

“Reflecting on social responsibility, during this emergency we are living as individuals and collective, is perhaps one of those few things that allow me to stay rooted to the ground. Social responsibility means to become aware and ‘force’ awareness even when our body or our mind would rather hide under the blanket with our eyes closed until the pandemic ends. It means to open the path to new ways of learning from, and changing, what we do”. (ME_B).

Finally, one participant stressed how the experience helped him understand the importance of advocating for relevant issues.

“Thanks to XE-SL, I realized that battles cannot be fought only in my head or at the bar with friends. There are places where concrete actions are implemented in the community. We stand up for it even with strangers, having the courage to say, “I’m not going to take this anymore””. (GR_C).

#### 3.1.2. Competence Awareness

Qualitative data showed participants’ reflection and awareness of deployed and learned competencies throughout the experience. Engaging with the community organizations’ activities and projects elicited different democratic competencies, namely students’ perspective-taking, personal beliefs, cultural background respect, and adaptability.

“I’ve learned that, to understand things deeply, you need to put yourself in others’ shoes. And that’s what I did. Doing so, I understood the complex reality of community organizations and that some of the things that I considered as constructive contributions [ameliorative suggestions] to the community might become work overload for community practitioners [require too many additional resources and competencies to be implemented]”. (AB_C).

“I learned how to approach and respect new people and especially people of other nationalities and cultures, realizing that my contribution can be useful to other people”. (CR_B).

“Coming from three different fields of study, in the beginning, our conversations [as XE-SL students] were complex, and sometimes I had the feeling that we did not understand each other fully, as if we spoke different languages. During the experience, conversations became clearer, and differences were no longer relevant. I think it was satisfying for everyone and helped us to create a positive team climate”. (AF_B).

Intertwined with democratic competencies, excerpts emphasized the group dimension and the related transferable teamwork and leadership competencies trained and strengthened during XE-SL. Overall, working in a group represented an opportunity to grow and learn how to better master group dynamics.

“I’ve learned the importance of reciprocity when working in a group. When we were all engaged positively, virtuous circles were established. This also motivated other students and community members to be committed and reciprocate”. (AF_B).

“From this [XE-SL] experience, I’ve learned the importance and beauty of collaborating with my XE-SL colleague. I believe that collaboration is a competence that will be very useful and fundamental in the future. I hope to feel so at ease with a future team in my job as well”. (VL_C).

“Sometimes, within a group, different ideas can emerge, and they might not always be perceived correctly by others. I think that the secret is to always talk about it and find compromises. By doing this, you can reach personal and collective growth”. (EL_C).

One participant found an additional meaning to teamwork during the challenging times of the pandemic. To her account, “In a period like this [the COVID-19 Italian lockdown], working together is not only a way to optimize work, but a human need” (ME_B).

Participants reported other transferrable competencies deriving from the experience. Students trained their communication competence, learning to present themselves in new contexts and overcoming shyness and sharing their needs.

“Being a shy person, XE-SL helped me to be more confident in meeting strangers. I had the chance to present things in front of an audience, talk to people I did not know, share my ideas and thoughts in the clearest way possible. In general, the university is helping me in this; but the support of XE-SL has been essential for me”. (LD_B).

“I’ve learned to share my needs with others and don’t leave space to the unsaid to build and maintain a good communication”. (AF_B).

Performing different activities and conveying personal competencies and passions toward XE-SL projects helped participants be aware of their creativity. One student realized how his passion for music could be used to create a product for children.

“The thing that surprised me the most was my creativity. I didn’t think I could create a story for children with visual disabilities [final product of the XE-SL project] like this”. (GR_C).

XE-SL offered students a guided experience inside organizational realities, practicing collaboration, time management, and, in a broader sense, organizational competencies.

“I have learned what it means to be there for others, listening and sharing opinions in a work context. I’ve also learned to work in a group, respect deadlines, and collaborate to contribute to individual and collective well-being”. (GA_B).

### 3.2. Sense of Responsibility and Democratic Competencies: Quantitative Data

Table 1 reports the descriptive statistics of the variables under inspection on the quantitative data: mean values, standard deviations, and correlations. Low SD values showed how the data are overall clustered around the mean. Mean values showed a general tendency of self-reported lower scores in the post-test in most of the considered variables—except for cultural background respect (CBR) and global mindedness (GM). This tendency can be explained by a response shift bias, which occurs when students overestimate their self-reported competencies at the time of the pretest and, through the course of a particular intervention, recognize that their actual competencies’ mastery is lower than what they initially reported [39].

Bivariate correlations with Spearman’s rho showed a tendency of fan-spread correlation change, that is, when a positive correlation is observed between initial status (i.e., precondition) and change (i.e., postcondition) [40]. Most pre variables were also significantly correlated to their post counterpart, except for CBR. We could find significant positive correlations between SOCR pre and GM pre (r = 0.738; *p* = 0.001), between SOCR pre and GM post (r = 0.498; *p* = 0.042), between SOCR post and GM pre (r = 0.559; *p* = 0.020), and between SOCR post and GM post (r = 0.601; *p* = 0.011). The significant positive correlation between these two constructs can be referred to as the social responsibility dimension that both scales evocate. Moreover, the data showed a general negative correlation between GM and adaptability (Ad) both in the pre (r = −0.544; *p* = 0.024) and post (r = −0.572; *p* = 0.016); and the negative correlations of sense of community responsibility (SOCR) with Ad pre (r = −0.490; *p* = 0.046) and post (r = −0.668; *p* = 0.003). This phenomenon might be explained by the fact that the scales are differentiated by internally cognitive (Ad) versus externally social (GM and SOCR) focuses [41]. In a way, the Ad construct echoes a cognitive process by which individuals are capable of adapting their thinking and behaviors according to the prevailing environment. On the contrary, GM and SOCR, for their social responsibility dimension, recall an agentic process toward changing the context for the better. Finally, another significant correlation is between CBR post and perspective-taking (PT) post (r = −0.547; *p* = 0.016). This negative correlation seems counterintuitive. According to the OECD framework [13] (p. 9), “engaging with different perspectives and world views requires individuals to examine the origins and implications of others’ and their own assumptions. Individuals with this competence also account for and appreciate the connections (e.g., basic human rights and needs, common experiences) that enable them to bridge differences and create common ground. Recognizing another’s position or belief is not necessarily to accept that position or belief. However, the ability to see through "another cultural filter" provides opportunities to deepen and question one’s own perspectives”. Following this perspective, we can speculate that a possible explanation lies in the fact that most students’ service activities were implemented with site supervisors and that this prevented them from experiencing "another cultural filter." A Wilcoxon signed-rank test showed that the XE-SL experience elicited a statistically significant change in the GM democratic competence in university students (Z = −3.143, *p* = 0.002). Indeed, the median global mindedness score rating was Mdn = 4.0 in the pre-survey and Mdn = 4.4 in the post-survey. No significant change was reported for other variables under examination (cf. Table 2).

### 3.3. Assets and Limits of XE-SL

The third research question was related to the students’ perceptions of XE-SL. Overall, qualitative data showed a greater focus on XE-SL assets rather than limits. Participants identified the reflexivity component of XE-SL as the most significant asset. To their account, reflection on the experience and the activities represented good practices to organize ideas and feelings, stressing that reflection processes are generally not stimulated elsewhere.

“Reflecting on this XE-SL experience allows me to ‘tidy up’ my ideas and feelings, understanding and discovering things about myself that maybe I already knew but I did not know how to express”. (AF_B).

“It was an exciting and fruitful experience. Throughout this course, I had the chance to learn and reflect on things and generally do something that I could hardly do otherwise”. (GA_B).

Moreover, the XE-SL experience allowed students to face negative feelings without giving up on the learning dimension, making them more aware of their beliefs and attitudes.

“I like to believe that everything can be solved for the better, that there is a good explanation for everything. This XE-SL experience was frustrating sometimes but made me realize that my way of thinking doesn’t make me see things realistically. But it’s important to see reality. Why and where a problem starts and how to solve it. Otherwise, we cannot solve problems. I hope this new habit of reflecting on things will continue to guide me in solving the problems that I will encounter in the future. With the awareness, this time, that I am not alone, even when everything seems to row against, and that what I can do, whatever it is, is important”. (MGS_B).

Connection was reported as another relevant asset for students. XE-SL enabled the connection and encounter of different community organizations’ realities that were sometimes unknown to participants even if they were territorially close to them.

“The XE-SL experience helped me get in touch with an organization in my neighborhood, of which I did not know the existence”. (TC_B).

Participants also stressed the relevance of engaging with the community as part of their academic training. They acknowledged that it does not take place very frequently, remarking how XE-SL allowed them to serve and be agents of change while playing the role of students.

“I don’t get in touch with the community frequently in my university course. XE-SL allowed me to serve the community, deploy my competencies, and enable social change as a student. I felt like an added value for the organization”. (LC_B).

Meeting new realities motivated participants to continue to be engaged even after the XE-SL experience, embracing their role as active citizens/agents of change and understanding the value of altruism.

“I will continue to join the organization’s activities. Now that I know how to engage with the community actively, I feel energized, and I hope to change things, even if little by little, for the better in my community. I feel like to be a more active and influential citizen in society. I feel more responsible and aware of the reality that surrounds me”. (DC_C).

“The energy that I perceived during the activities, despite being online, made me understand the importance of XE-SL and, more generally, of altruism. How it can be an advantage both for the community and for the individuals being part of it”. (AF_B).

“This experience enriched me as a human. I still think that I do and commit myself too little. I would like to be more engaged and committed to my community. I would like to continue to serve this [XE-SL] association or at least devote my time to other associations. For example, I know very well a community organization in my city and, even if I had the opportunity, I never gave my contribution. Probably because I was afraid of not having specific skills. This XE-SL activity made me realize that commitment is precious and that everyone has some skill to offer”. (CR_B).

The experience also represented a way to “learn more and more about online meetings” (AF_B), and become acquainted with new applications thanks to the help of others.

“I learned how to use a new social media application. The site supervisors spent time explaining how the application worked and all its different functions”. (LD_B).

According to participants’ reflexive journals, the main limitation was the online dimension of the experience. The virtual connection prevented students from being in-presence, sharing places, and took away "some of the magic" of living in the community spaces. Nevertheless, students also acknowledged the potential of being online to feel less alone, still be productive, be somehow together, and actively collaborate.

“Doing it from home maybe took away ‘some of the magic’ of being in-presence. Still, it allowed us to join a close-knit group and actively collaborate”. (TC_B).

“I think that the online dimension limits the experience. However, it is the only way to keep us together today, be productive, and feel less alone. Still, I think it distracts us from reality”. (VL_B).

## 4. Discussion

The COVID-19 pandemic led governments worldwide to swiftly implement measures (e.g., school closure, social distancing, lockdown) to reduce disease transmission. This transition meant taking lectures and student activities from face-to-face mode to online teaching and learning [2].

This study shed insights into the XE-SL experiences of Italian university students during the COVID-19 lockdown and is unique for several reasons. First, a scarcity of studies investigated the impact of university students’ learning experience under COVID-19. Second, few studies focused on XE-SL courses, which entails a strong experiential component. Third, only few of the existing studies adopted validated assessment tools or a mixed-method approach akin to the present study. Lastly, while some research investigated the sense of responsibility dimension during the pandemic in Italy and the engagement with the community [42], there are no published studies on SL Italian experiences during the COVID-19 national quarantine and their impact on the sense of responsibility.

The study, indeed, aimed to explore the responsibility and democratic dimensions elicited by the XE-SL experience and the perceptions of engaging in exclusive online service activities with local communities that were inaccessible elsewhere. To analyze these aspects, we conducted a qualitative-driven mixed-method study. Students’ reflexive journals and pre-post surveys were used to collect data.

Regarding the first research question (RQ1), the findings suggest that XE-SL had a role in enhancing students’ sense of responsibility toward the community, representing an eye-opening experience for students, especially during the COVID-19 lockdown situation. It helped participants become aware of local organizations and their mission, being part of the change and bringing new perspectives to organizations while understanding the relevance of standing up and advocating for relevant issues. These dimensions of responsibility corroborate previous findings [20] and are closely connected to social justice promotion, related to XE-SL and SL experiences [43,44]. Quantitative data ambivalently added to qualitative findings. As presented before, the GM scale embedded a responsibility declination and was the only dimension to grow significantly after the experience. Vice versa, SOCR was one of the dimensions where students scored, nonsignificantly, lower in the post-administration than the pre. One possible explanation is provided by the response shift bias suggested by Rohs and Langone [39]. Qualitative data reinforced this interpretation, stressing the reflection activity that students went through that might help participants be more aware of what it truly means to “be there for the community” even when costs are greater than the benefits.

For the second research question (RQ2), qualitative data offered a clear perspective that XE-SL was a place to strengthen democratic competencies and learn and deploy new ones. The findings also disclosed the reflection of other transferable competencies (i.e., teamwork, leadership, communication, creativity, and organizational competencies) that are not strictly related to the democratic dimension. These findings confirm and add to the existing literature on the role of XE-SL, and eSL in general, to foster transferable competencies such as teamwork [10,19,45]. Quantitative data did not remark this finding and presented a general, though not significant, decrement in scores in post variables—except for CBR that had an increment in the post-administration, yet not statistically significant. Besides embedding a responsibility declination, the GM democratic competence made the only exception. This finding corroborates previous studies that identified the global dimension emerging from the data collected with students as one of the main effects of XE-SL and eSL experiences [10]. This phenomenon may lay in the nature of the XE-SL experiences, where students were boundlessly connected to communities online; this might help them open up and reflect more globally instead of locally. The COVID-19 pandemic may also have played a role, casting greater attention on global perspectives and making explicit the global interconnectedness in which we all live (e.g., by showing how things that take place in other countries inevitably impact our country and our daily lives).

Regarding the third research question (RQ3), qualitative data showed that students tend to reflect on the positive aspects of XE-SL and what they can do rather than focus on the limits. XE-SL assets can be divided into three main categories. The first is “reflection,” perceived as a good practice to be integrated into everyday life, given its capability to raise awareness on things and processes. Reflecting on the learning and the service-empowered students to assess individual learning goals while collaborating with others to make meaning of their XE-SL experience, and acknowledging the shared/cooperative dimension of the learning process. This finding stresses the central role of reflection in eSL and SL courses [44,46]. The second dimension is “connection,” with distant and proximal realities. This dimension echoes the sense of community construct, first introduced by McMillan and Chavis [47]. According to the authors, sense of community is related to a sense of belonging, connection, and interdependence of individuals within a community. Sense of community is recognized as a catalyst and a correlate for civic engagement and participation. In this case, we can conclude that XE-SL positively influenced students’ sense of community even if the civic engagement activities took place exclusively online. The last dimension is “change for the community,” which can be produced as students, as citizens, and as future practitioners, emphasizing the students’ role in assisting communities in finding solutions to their main challenges [23].

About perceived limits, participants did not explicitly and univocally address this aspect. Nevertheless, adapting face-to-face SL experiences to exclusively online activities evoked ambivalent feelings in students and provided valuable opportunities for students to serve communities in diverse ways while enhancing their online and social media competencies and literacy [48].

On the one hand, they acknowledged the possibility of being agents of change and answering community needs while gaining new and strengthened competencies. On the other hand, the online dimension prevented them from fully experiencing the situated and territorial dimension of communities and, therefore, can be presented as the major limit to the XE-SL experience. The COVID-19 pandemic, and the national quarantine, required students to face their own personal challenges in their communities and social systems. Moreover, students had to confront the abrupt shift to online forms of learning and civic engagement all at once. We can hypothesize that these factors also played a central role for some participants in perceiving the XE dimension of SL as a limit, especially during the first stages of the XE-SL experience, as students may have lacked time to internalize what was taking place around them.

## 5. Conclusions

The study shed a positive light on the capability of SL in its extreme online form to promote a sense of responsibility, civic engagement, and the acquirement of democratic and transferrable competencies, such as perspective-taking, adaptability, cultural background respect, global mindedness, teamwork, leadership, communication, creativity, and organizational competencies.

The reflection and connection promoted and sustained by the XE-SL are fundamental aspects for participants that perceive them as added values for their academic, personal, and future professional lives.

The use of a mixed-method and longitudinal pre-post data collection enabled data triangulation. It also deepened the understanding of phenomena, i.e., to formulate the response shift bias effect hypothesis on the nonsignificant decrement in some variables taken under inspection in the quantitative study.

A question remains open. Would students stick to the XE-SL format if not forced to stay home by the national lockdown?

The qualitative excerpts stressed that the central perceived limit was the exclusive online dimension of the experience. This result might have been influenced by the frustration of the pandemic experience of being forced to stay home. In a way, every form of online experience might have been perceived as limited in a time when connections could only take place online. The findings suggest that the extreme online form is not suited for everyone or every situation but that more hybrid forms of eSL could be more engaging and perceived as less limiting. On this, instructors and community partners (or site supervisors) can play a relevant role in designing XE-SL courses that foresee the possibility of including face-to-face service activities when required (e.g., when the XE dimension is perceived as limiting by students), thus underpinning a good quality of participation, and fostering student engagement [49,50]. To achieve that, effective communication should be established between instructors/site supervisors and students to promote positive emotions, that play a central role in distance education, within the learning process [51].

This study had some limitations. The main limitation was the small sample size. Having twenty students enrolled in this XE-SL course also represented a positive thing for the SL academic staff and the community partners. Students decided to be engaged with the communities no matter what. Nevertheless, having a small sample prevented us from computing more sophisticated quantitative analysis and generalizing results.

Our study contributes to the existing literature by adding further responsibility and democratic benefits of XE-SL experiences. The dissonant findings underline the relevance of adopting a mixed-method design to approach SL experiences to interpret students’ experiences better and implement XE-SL (and SL) practice. Moreover, the quality of participation and ownership dimensions should be incorporated as relevant eSL elements in nurturing a positive development of students’ sense of responsibility and democratic competencies.

Our study also suggests some practical implications. The use of online technology entails the possibility of overcoming physical restrictions and offering more inclusive learning to students, but the lack of the on-site component is perceived as “the missing piece” of the experience. Therefore, more structured activities are suggested to ease the interaction and connection with community organizations and NGOs. A way to favor this would be to rethink the timeframe of XE-SL courses, adjusting the number of total service hours accordingly to the SL form—that is, face-to-face SL, hybrid eSL, and XE-SL.

This study also opens to future research on XE-SL experiences in nonemergency contexts, comparing XE-SL to eSL and SL experiences with a longitudinal mixed-method design, understanding eventual significant differences, or corroborating existing literature that claims no differences [17]. This would deepen the understanding of the role of exclusive online interactions required by XE-SL for participants. Moreover, further studies with a larger sample would disentangle the reflection over the adequacy of the OECD questionnaire to assess the change in students’ democratic and global competencies within XE-SL—and perhaps face-to-face SL—experiences.

To conclude, our study proves the goodness and efficacy of XE-SL in sustaining and fostering students’ sense of ownership and responsibility, even in the hard times of the COVID-19 national quarantine. XE-SL allowed students to experience and live community contexts, standing (virtually) together by staying (physically) apart.

## Figures and Tables

**Table 1:** Descriptive statistics.

		M	SD	1	2	3	4	5	6	7	8	9	10	11
1	PT Pre	2.06	0.60	-										
2	PT Post	2.02	0.57	0.526 *	-									
3	Ad Pre	2.30	0.50	0.443	0.269	-								
4	Ad Post	2.15	0.68	0.258	0.465	0.688 **	-							
5	CBR Pre	4.65	0.43	−0.041	−0.115	−0.352	−0.157	-						
6	CBR Post	4.80	0.28	−0.225	−0.574 *	−0.048	−0.344	0.013	-					
7	GM Pre	3.81	0.67	−0.375	−0.196	−0.544 *	−0.576 *	0.062	−0.003	-				
8	GM Post	4.19	0.76	−0.262	−0.282	−0.439	−0.572 *	0.101	0.286	0.753 **	-			
9	SOCR Pre	3.34	0.62	−0.117	−0.038	−0.490 *	−0.455	0.023	−0.094	0.738 **	0.498 *	-		
10	SOCR Post	3.27	0.50	−0.011	−0.180	−0.430	−0.668 **	0.032	0.166	0.559 *	0.601 *	0.803 **	-	
11	PBI Pre	2.36	1.20	0.238	0.322	−0.005	0.135	−0.013	0.059	0.086	−0.017	0.249	−0.007	-
12	PBI Post	2.32	1.15	0.261	0.259	0.189	0.288	−0.095	0.029	0.073	−0.014	0.265	0.042	0.784 **

* The correlation is significant at the 0.05 level (2-tailed). ** The correlation is significant at the 0.01 level (2-tailed). M = Mean; SD = Standard Deviation; PT = Perspective-Taking; Ad = Adaptability; CBR = Cultural Background Respect; GM = Global Mindedness; SOCR = Sense of Community Responsibility; PBI = Personal Beliefs about Inequalities.

**Table 2:** Wilcoxon signed-rank test values.

	Z	*p*
Perspective-Taking	−0.287	0.774
Adaptability	−1.074	0.283
Cultural Background Respect	−1.069	0.285
Global Mindedness	−3.143	0.002
Sense of Community Responsibility	−1.048	0.295
Personal Beliefs	−0.342	0.732

## Data Availability

The data that support the findings of this study are not publicly available, due to privacy restrictions. They are available from the corresponding author upon reasonable request.
